# Analysis and Prediction of Subway Tunnel Surface Subsidence Based on Internet of Things Monitoring and BP Neural Network

**DOI:** 10.1155/2022/9447897

**Published:** 2022-05-14

**Authors:** Baitian Wang, Jing Zhang, Longhao Zhang, Shi Yan, Qiangqiang Ma, Wentao Li, Maopeng Jiao

**Affiliations:** ^1^School of Architecture and Civil Engineering, Shangqiu University, Shangqiu 476113, Henan, China; ^2^School of Civil Engineering, Nanjing Tech University, Nanjing 210000, Jiangsu, China; ^3^Department of Civil and Environmental Engineering, Rensselaer Polytechnic Institute, Troy, NY 12180, USA; ^4^Huludao Highway Survey and Design Institute Co. Ltd., Huludao 125003, China; ^5^Modern Educational Technology Center, Mudanjiang Medical University, Mudanjiang, Heilongjiang 157011, China; ^6^School of Civil Engineering, Qingdao University of Technology, Qingdao 266033, China; ^7^School of Civil Engineering, Southeast University, Nanjing 211189, China

## Abstract

With the acceleration of the urban development process and the rapid growth of China's population, the subway has become the first choice for people to travel, and the urban underground space has been continuously improved. The subway construction has become the focus of urban underground space development in the 21st century. During the construction of subway tunnels, the problem of surface settlement will inevitably be caused, and the problem of surface settlement will have a certain safety impact on the safe use of surface buildings. The impact of surface construction is predicted, so as to select the best construction technology and avoid the problem of surface subsidence to the greatest extent. On the basis of analyzing the principle of surface subsidence, this paper studies the optimal control strategy and process of subsidence in subway tunnel engineering. The research results of the article show the following. (1) The two sections of the pebble soil layer have basically the same subsidence trend. Among them, the first section has a larger settlement amplitude and both sides are steeper. The second section is mainly cobble clay soil. The pebble layer has good mechanical properties. If it can be well filled, its stability will be improved. The comparative analysis of the two sections shows that with the increase of the soil cover thickness, the maximum subsidence at the surface gradually decreases. The reason is that when the stratum loss is the same, the greater the soil cover thickness, the greater the settlement width. Sections 2 and 3 of a single silty clay have relatively close settlement laws, and the settlement changes on both sides of the tunnel are similar. (2) The surface subsidence caused by the excavation of the side hole accounts for more than 50% of the total surface subsidence, and the width of the settlement tank after the excavation of the side hole is increased by 8–10 meters compared with the excavation of the middle hole. (3) The prediction error of the BP neural network model proposed in this paper is the lowest among the four models, whether it is the prediction of the cumulative maximum surface subsidence or the location of the cumulative maximum surface subsidence, and the average relative error of the cumulative maximum surface subsidence is 3.27%, the root mean square error is 3.87, the average relative error of the location of the cumulative maximum surface subsidence is 7.96%, and the root mean square error is 21.06. In the prediction process of the cumulative maximum surface subsidence, the prediction error value of the Elman neural network is relatively large, and the GRNN generalized neural network and RBF neural network have no significant changes; in the process of predicting the position where the cumulative maximum surface subsidence occurs, the prediction error value of RBF neural network is maximum.

## 1. Introduction

With the acceleration of urban development, our country's subway construction has also entered a period of rapid development. In order to meet the convenience of the general public, subway tunnels often pass through the city center. The impact of subway construction on the surface may lead to the safety and use of surface buildings. In order to avoid and eliminate this impact as much as possible, we must predict the impact of subway construction on surface buildings, so as to choose the best construction technology that can avoid the problem of surface subsidence to the greatest extent. There are many factors that affect the surface subsidence. In the process of subway construction, the problem of surface subsidence is of great concern. This article will analyze and study the law of subway subsidence and make predictions of surface subsidence. Hui-Jun and Heng [1] analyzed the deformation law and the influence of construction sequence on the surface subsidence based on the monitoring and measurement of the surface subsidence. Wang et al. [2] took the Wuhan Metro Line 3 project as the background, adopted the method of combining on-site monitoring and numerical simulation, and compared and analyzed the vertical surface subsidence law. Li and Shaw [3] analyzed and summarized the methods of controlling the subsidence of borehole shallow tunnels under the surface structure under the background of engineering design and construction. Hui [4] took the Chehuang Station section of the northern extension of Guangzhou Metro Line 4 as the engineering background and analyzed the surface subsidence caused by the main construction parameters of the shield tunnel. Jie et al. [5] took the whole area parallel to the tunnel as an example and analyzed the influence of five basic calculations. Yu and Province [6] combined the case of surface settlement of Wuhan Line 7 shield tunnel excavation and used mathematical linear analysis and fitting to correct the formula according to its geological conditions and measurement data. Wei [7] took Lanzhou subway tunnel as the engineering background and used GTS-NX software to simulate the construction process. Han [8] analyzed the longitudinal and lateral development and distribution of surface subsidence caused by single and double excavation based on Xi'an Metro Line 1. Xiao-Ping [9] verified the reliability of construction and monitoring in subway construction and provided a reference for subway design and construction. Li et al. [10] took the shield construction of Dalian Metro 202 bid section as an example to monitor and measure the surface settlement and top surface settlement during the shield construction process. Pei et al. [11] used the control variable method to analyze the monitoring data of the construction process and summarized the law of surface settlement. Wang et al. [12], after 3 months of accuracy level measurement, showed that the monitoring results have important guiding and reference significance for the construction of Dalian subway and similar shallow tunnels. Bo [13] applied the deep hole grouting technology to expound the reliability of construction and monitoring technology in subway construction and provided a reference for subway construction. Duan et al. [14] proposed a Gaussian equation based on the actual measurement in a limited area, which is to predict the soft soil movement caused by shallow tunneling. In [15], in order to study the surface subsidence of Dalian Metro Section 202, a monitoring and measurement station was built during the construction process.

## 2. Analysis and Prediction of Surface Settlement of Subway Tunnels

### 2.1. Construction Method of Subway Tunnel

With the continuous improvement and development of subway construction technology, tunnel engineering technology has become more and more abundant, and urban construction methods can generally be divided into three categories. The details are shown in Table 1.

### 2.2. Factors Affecting Surface Subsidence

According to a large number of engineering practices, there are many factors that affect the surface settlement. Different construction methods, geographical conditions, thickness of covering soil, etc. will affect the surface settlement during tunnel construction. The factors affecting land subsidence are not isolated but interact with each other. The optimal design of construction is inseparable from the professional quality and work level of the construction personnel. In the specific construction process, the staff should check the layers, comprehensively analyze the influence of multiple factors, and take effective engineering measures to reduce the impact of construction as much as possible.

## 3. Analysis and Prediction of Surface Subsidence

### 3.1. Analysis of Surface Subsidence

Since tunnel excavation is a plane strain problem, according to the stochastic theory, the final ground subsidence _*e*_*W*(*X*) and surface horizontal displacement *U*_*e*_(*X*) at the distance *X* from the center of the unit caused by unit excavation are [19]

(1) $$\begin{array}{c} W_{e}\left(X\right)=\frac{\tan\;\beta }{\eta }\exp\left[-\frac{\pi \;\tan^{2}\;\beta }{\eta^{2}}X^{2}\right]\text{d}\varepsilon \text{d}\eta ,\\ U_{e}\left(X\right)=\frac{X\;\tan\;\beta }{\eta^{2}}\exp\left[-\frac{\pi \;\tan^{2}\;\beta }{\eta^{2}}X^{2}\right]\text{d}\varepsilon \text{d}\eta , \end{array}$$

where *β* is the main influence angle of the upper stratum of the tunnel, *O* − *εη* is the coordinate system adopted by the excavation unit rock and soil mass, and *O* − *XYZ* is the coordinate system adopted by the ground surface.

The surface settlement *W*(*X*) and the surface horizontal displacement *U*(*X*) caused by the excavation of the tunnel are, respectively [20],

(2) $$\begin{array}{c} W\left(X\right)=\int \int_{\Omega -\omega }\frac{\tan\;\beta }{\eta }\exp\left[-\frac{\pi \;\tan^{2}\;\beta }{\eta^{2}}{\left(X-\varepsilon \right)}^{2}\right]\text{d}\varepsilon \text{d}\eta ,\\ U\left(X\right)=\int \int_{\Omega -\omega }\frac{\left(X-\varepsilon \right)\tan\;\beta }{\eta^{2}}\exp\left[-\frac{\pi \;\tan^{2}\;\beta }{\eta^{2}}{\left(X-\varepsilon \right)}^{2}\right]\text{d}\varepsilon \text{d}\eta . \end{array}$$

According to the replacement formula of double integral, we can get

(3) $$\begin{array}{c} \int \int_{D}f\left(\varepsilon ,\eta \right)\text{d}\varepsilon \text{d}\eta =\int \int_{D^{\prime }}f\left[X_{1}+r\;\cos\;\theta ,Z-r\;\sin\;\theta \right]r\text{d}r\text{d}\theta . \end{array}$$

Subsidence value:

(4) $$\begin{array}{c} W\left(X\right)=\int_{r_{1}}^{r_{2}}\int_{\theta_{1}}^{\theta_{2}}\left\{\frac{\tan\;\beta }{Z_{1}-r\;\sin\;\theta }\cdot \;\exp\;{\left[X-\left(X_{1}+r\;\cos\;\theta \right)\right]}^{2}\right\}r\text{d}r\text{d}\theta . \end{array}$$

Surface level displacement:

(5) $$\begin{array}{c} U\left(X\right)=\int_{r_{1}}^{r_{2}}\int_{\theta_{1}}^{\theta_{2}}\frac{\left[X-\left(X_{1}+r\;\cos\;\theta \right)\right]\tan\;\beta }{{\left(Z_{1}-r\;\sin\;\theta \right)}^{2}}\cdot \;\exp\left\{-\frac{\pi \;\tan^{2}\;\beta }{{\left(Z_{1}-r\;\sin\;\theta \right)}^{2}}\left({\left[X-\left(X_{1}+r\;\cos\;\theta \right)\right]}^{2}\right\}\right\}r\text{d}r\text{d}\theta . \end{array}$$

Surface subsidence and deformation formula:

(6) $$\begin{array}{c} W\left(X\right)=\sum_{i=1}^{n}W_{i}\left(X\right),\\ U\left(X\right)=\sum_{i=1}^{n}U_{i}\left(X\right). \end{array}$$

Surface subsidence and surface horizontal displacement caused by the construction of the double-hole circular tunnel [21]:

(7) $$\begin{array}{c} W\left(X\right)=W_{1}\left(X\right)+W_{2}\left(X\right),\\ U\left(X\right)=U_{1}\left(X\right)+U_{2}\left(X\right). \end{array}$$

Horizontal deformation:

(8) $$\begin{array}{c} E\left(X\right)=\frac{\text{d}U_{1}\left(X\right)}{\text{d}X}+\frac{\text{d}U_{2}\left(X\right)}{\text{d}X}. \end{array}$$

Tilt:

(9) $$\begin{array}{c} T\left(X\right)=\frac{\text{d}W_{1}\left(X\right)}{\text{d}X}+\frac{\text{d}W_{2}\left(X\right)}{\text{d}X}. \end{array}$$

Curvature:

(10) $$\begin{array}{c} K\left(X\right)=\frac{d^{2}W_{1}\left(X\right)}{\text{d}X^{2}}+\frac{d^{2}W_{2}\left(X\right)}{\text{d}X^{2}}. \end{array}$$

### 3.2. Prediction Model Establishment

The mathematical model of the settlement tank of the tunnel construction surface cross section follows the normal distribution curve, and the calculation formula of the settlement tank is

(11) $$\begin{array}{c} s=s_{\max}\exp\left(\frac{-y^{2}}{2i^{2}}\right], \end{array}$$

where *y* is the distance from the centerline of the tunnel, *s* is the surface subsidence at *y* from the centerline of the tunnel, *s*_max_ is the maximum subsidence at the centerline of the tunnel, and *i* is the distance from the inflection point to the centerline of the tunnel.

It can be concluded that

(12) $$\begin{array}{c} \ln\left(\frac{s}{s_{\max}}\right)=\frac{-1}{2i^{2}}y^{2}. \end{array}$$

Settling tank width parameters:

(13) $$\begin{array}{c} i=\sqrt{\frac{1}{2m}}. \end{array}$$

The formula fitting of the single-hole settlement tank [22]:

(14) $$\begin{array}{c} s=16.5\;\exp\left(\frac{-y^{2}}{2\times 9.53^{2}}\right]. \end{array}$$

Shuangdong surface subsidence prediction model [23]:

(15) $$\begin{array}{c} s=s_{L}+s_{R}=s_{R\max}\exp\frac{-{\left(y+y0\right)}^{2}}{2i_{R}^{2}}+s_{L\max}\exp\left(\frac{-{\left(y-y0\right)}^{2}}{2i_{L}^{2}}\right]. \end{array}$$

The right hole settlement tank formula fitting:

(16) $$\begin{array}{c} s_{R}=16.5\;\exp\left(\frac{-{\left(y+7.5\right)}^{2}}{2\times 13.13^{2}}\right]. \end{array}$$

The formula fitting of the settlement tank in the left hole:

(17) $$\begin{array}{c} s_{L}=27.2\;\exp\left(\frac{-{\left(y-7.5\right)}^{2}}{2\times 13.13^{2}}\right]. \end{array}$$

The total settling tank formula [24]:

(18) $$\begin{array}{c} s=s_{R}+s_{L}=16.5\;\exp\frac{-{\left(y+7.5\right)}^{2}}{2\times 9.53^{2}}+27.2\;\exp\left(\frac{-{\left(y-7.5\right)}^{2}}{2\times 13.13^{2}}\right]. \end{array}$$

## 4. Simulation Experiments

### 4.1. Measured Data Analysis

During construction, in addition to having a certain impact on the axis of the excavation surface, it may also have a certain impact on the surrounding environment. In order to increase the reliability of the experiment, the experiment selected different sections of the subway tunnel for research. There are subway tunnels dominated by pebble soil layers and subway tunnels dominated by single silty clay.

#### 4.1.1. Pebble Soil Layer

**Figure 1:**
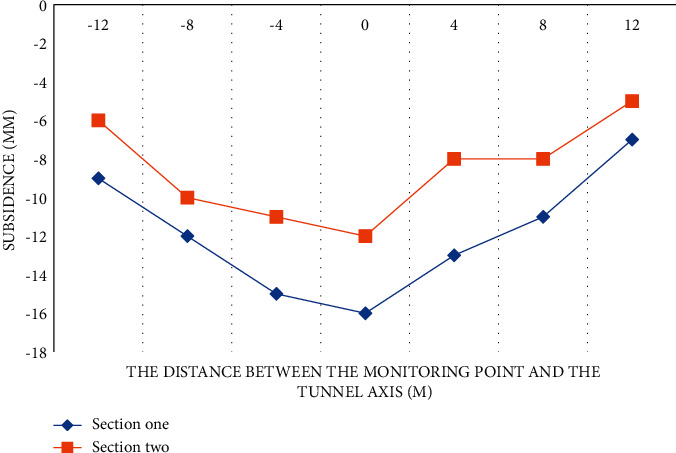
Construction settlement curve of pebble soil layer.

The subway tunnel in the pebble soil layer is buried about 14 meters deep, and two sections were selected for the experiment. Section 1 is mainly coated with medium and fine sand at the position of shield tunneling, and the upper and lower layers are both pebble layers. Section 2 is buried at a depth of about 15 m. The shield tunneling is mainly composed of pebble layers, and the upper and lower layers are all gravel and silty clay. The construction settlement curve of the pebble soil layer is shown in Figure 1.

According to the data in Figure 1, we can conclude that the two sections have basically the same subsidence trend. Section 1 has a larger subsidence amplitude and both sides are steeper. The main reason is that the stratum where Section 1 is located is mostly fine sand and pebbles. Based on the discontinuous particle distribution, quicksand and other conditions may occur during construction, resulting in a large amount of settlement. This is a typical shallow buried deep section of a shield tunnel, the settlement curve is relatively steep, and there is a large settlement amount. Section 2 is mainly cobble cohesive soil. The pebble layer has better mechanical properties. If it can be well filled, its stability will be better than that of the sand layer. The pebble layer has good water permeability, and the groundwater level in the area where this section is located is low, and the water pressure during construction has little effect on the surface subsidence, so the subsidence curve displayed at the second section of the section has a relatively gentle variation. The comparative analysis of the two sections shows that with the increase of the soil cover thickness, the maximum subsidence at the surface gradually decreases. The reason is that when the stratum loss is the same, the greater the soil cover thickness, the greater the settlement width.

#### 4.1.2. Single Silty Clay

**Figure 2:**
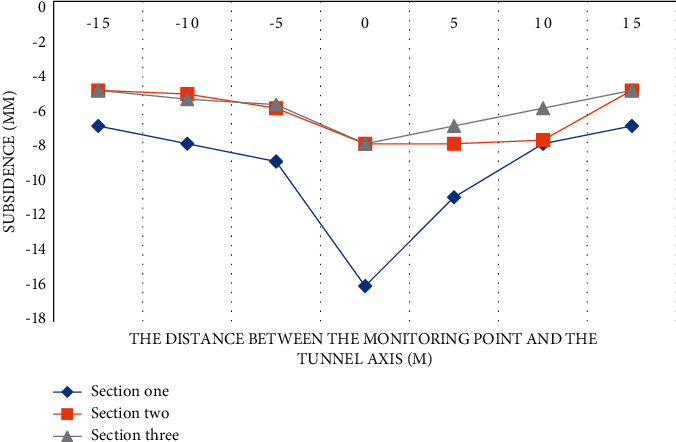
Construction settlement curve of single silty clay.

A single silty clay subway tunnel is about 14 m deep [25]. Moreover, the subway tunnel is located in the main city area, so it is greatly affected by external influences during the construction process. A total of 3 typical cross-sections are selected for research in this interval, and the results are shown in Figure 2.

From the experimental data in Figure 2, we can conclude that the second and third sections have relatively close settlement laws, and the settlement changes on both sides of the tunnel are similar. The tunnel is mainly distributed with cohesive soil layer within the diameter range, and the geological self-stability is good, so it is not easy to collapse during construction. Due to the better compaction performance, the soil layer is less disturbed during construction in the cohesive soil environment. The settlement value at one section of the section is larger, and the settlement changes on both sides are larger. This section is mainly distributed with thick miscellaneous fill layer, and the stability of surrounding rock is poor. On the whole, Section 1 and Section 2 can better reflect the settlement law of this interval.

To sum up, it can be seen from the above analysis that the mechanical properties of the sand and pebble stratum are poor, and they are greatly affected during construction and are prone to instability of the excavation surface. When the shield machine is passing through this kind of soil layer, if it encounters pebbles with a large particle size, it is necessary to ensure that the thrust of the shield machine is large enough to push it forward. However, the construction speed will be reduced at this time, and the impact on the surrounding environment will be increased. The sand layer has more coarse sand particles, the thrust increases significantly, and the rotation speed of the cutterhead increases significantly, so the impact on the soil layer during construction is more significant.

### 4.2. Analysis of Surface Subsidence

**Figure 3:**
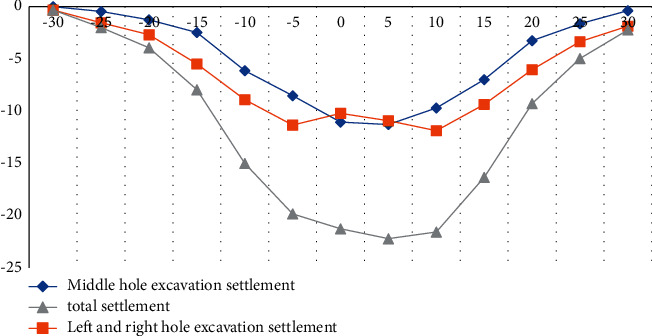
Lateral curve of surface subsidence in Section 1.

**Figure 4:**
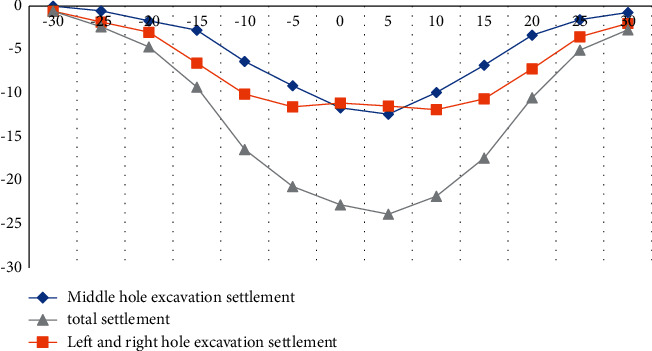
Lateral curve of surface subsidence in Section 2.

**Figure 5:**
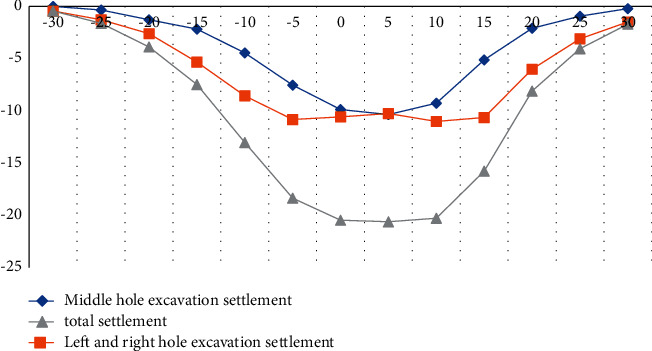
Lateral curve of surface subsidence in Section 3.

According to a large number of engineering practices and related literatures, there are many factors affecting the surface settlement. According to the research direction of the article, three different sections were selected for the experiment. Surface subsidence is caused by excavation. The experiment records the surface subsidence data of three measurement sections, as shown in Figures 3–5.

According to the above experimental data, we can conclude that during the excavation of the left and right side holes, the surface subsidence curve presents a wave shape, the wave trough is on the center line of the left and right lines, the wave crest is on the central axis of the structure, and the surface subsidence occurs during the excavation of the right side hole. It is larger than the left hole, mainly because when the left hole is excavated first, it will affect the bottom layer on the right side and cause geological softening. The surface subsidence caused by the excavation of the side hole accounts for more than 50% of the total surface subsidence, and the width of the settlement tank after the excavation of the side hole is increased by 8–10 meters compared with the excavation of the middle hole, as shown in Tables 2–4.

### 4.3. Model Checking

**Figure 6:**
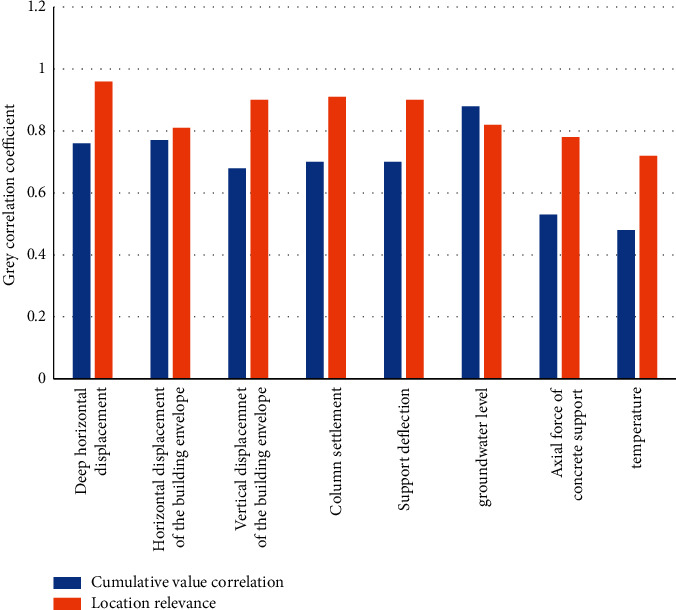
Correlation between monitoring content and surface subsidence.

Different network structures have different performances in predicting surface subsidence. In order to verify the good fitting accuracy and generalization ability of the BP neural network model, the BP neural network proposed in this paper is compared with the GRNN generalized neural network, RBF neural network, and Elman neural network to verify the effectiveness of the BP artificial neural network in surface subsidence prediction. Because the correlation of the experimental detection is not clear, the quantitative relationship between the monitoring content and the surface subsidence should be determined before the formal experiment, as shown in Figure 6.

**Figure 7:**
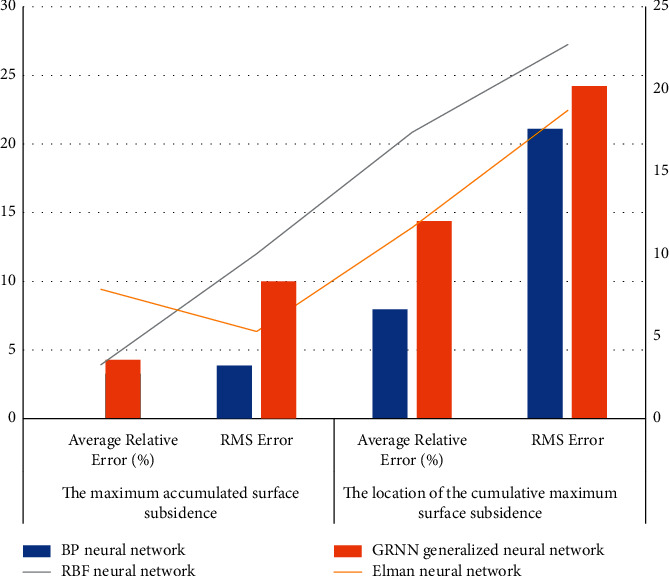
Statistics of accuracy and mean square error.

According to the data in Figure 6, we can conclude that among all the monitoring contents, the correlation between temperature and concrete support axial force and the surface settlement is the smallest, and the other monitoring contents have strong correlation with the surface settlement. It is almost the same as the position correlation sequence, so it can be seen that the grey correlation analysis can effectively evaluate the correlation between each monitoring content and the surface subsidence. Therefore, in order to avoid adverse effects on the generalization ability of the prediction model, the two monitoring contents of temperature and concrete support axial force are eliminated in this example, and the deep horizontal displacement, the vertical displacement of the enclosure structure, the horizontal displacement of the enclosure structure, and the settlement of the column are used. The supporting deflection, supporting axial force, and groundwater level are used as input variables to predict the surface settlement of foundation pit. We record the relative accuracy value and variance of each model, as shown in Table 5 and Figure 7.

According to the data in Table 5, we can conclude that the prediction error of the BP neural network model proposed in this paper is the lowest among the four models, whether it is the prediction of the cumulative maximum surface subsidence or the prediction of the location of the cumulative maximum surface subsidence. The average relative error of the cumulative maximum value is 3.27%, and the root mean square error is 3.87. The average relative error of the location of the cumulative maximum surface subsidence is 7.96%, and the root mean square error is 21.06. In the prediction process of the cumulative maximum surface subsidence, the prediction error value of the Elman neural network is relatively large, and the GRNN generalized neural network and RBF neural network have no significant changes; in the process of predicting the position where the cumulative maximum surface subsidence occurs, the prediction error value of RBF neural network is maximum. On the whole, the BP neural network model has the best training accuracy and generalization ability for the cumulative maximum surface subsidence and location, and its performance is better than the other three models. It shows that the initial weight and threshold search of BP neural network by artificial bee colony can prevent the network from falling into the local optimal value and effectively improve the network prediction ability.

## 5. Conclusion

In order to meet the travel problems of the general public and improve the utilization of urban underground space, there are more and more problems in tunnel construction. In the process of urban subway construction, it will inevitably have a certain impact on the rock and soil mass, and the geological deformation may affect the safety of building facilities on the ground. Therefore, the problem of surface subsidence caused by tunnel construction has always been a concern of many researchers. In order to effectively prevent and reduce the problem of surface subsidence caused by subway construction, it is necessary to make accurate predictions of the law of surface subsidence. The BP neural network prediction model proposed in this paper can accurately predict the subsidence law of the surface, and the prediction accuracy is as high as 90%. Since the research on the law of surface subsidence in this paper is based on the real side of surface data, the data obtained are not sufficient and have certain regional limitations. Therefore, in the future research work, data should be widely collected, and various methods should be used to predict and analyze the surface subsidence.

## Figures and Tables

**Table 1:** Comparison of several construction methods.

Comparison indicator	Cut-and-cover method [16]	Shield method [17]	Shallow burial and underground excavation method [18]
Geology	Various strata available	Various strata available	Water-bearing formations require special treatment
Place	Occupies more road area	Takes up less road area	Takes up less road area
Section change	Good adaptability	Poor adaptability	Good adaptability
Buried position	Shallow burial	Need some depth	Shallow burial
Waterproof construction	Easier	Easy	Difficult
Subsidence	Smaller	Smaller	Larger
Traffic obstacle	Greater impact	Less affected	Less affected
Underground pipeline	Demolition and protection required	No demolition and protection required	No demolition and protection required
Construction noise	Big	Small	Small
Surface demolition	Big	Small	Small
Water treatment	Precipitation, drying	Block and drop combination	Block, drop, or block-drain combination
Schedule	Affected by demolition and relocation, and the total construction period is relatively fast	The preliminary project is complex and the total construction period is average	Fast start and slow total construction period

**Table 2:** Surface subsidence during excavation of each part of Section 1.

Distance from midline	Middle hole excavation settlement	Excavation settlement of left and right side holes	Total settlement	Middle cave settlement/total settlement	Side tunnel settlement/total settlement
−25.6	0	−0.32	−0.32	0.0	100.0
−20.6	−0.46	−1.53	−1.99	23.1	76.9
−15.6	−1.25	−2.68	−3.93	31.8	68.2
−10.6	−2.47	−5.47	−7.94	31.1	68.9
−5.6	−6.12	−8.89	−15.01	40.8	59.2
−3.6	−8.51	−11.33	−19.84	42.9	57.1
−1.6	−11.05	−10.21	−21.26	52.0	48.0
1.6	−11.28	−10.92	−22.20	50.8	49.2
3.6	−9.69	−11.87	−21.56	44.9	55.1
5.6	−6.98	−9.35	−16.33	42.7	57.3
10.6	−3.26	−6.02	−9.28	35.1	64.9
15.6	−1.63	−3.34	−4.97	32.8	67.2
20.6	−0.37	−1.86	−2.23	16.6	83.4
25.6	0	−0.49	−0.49	0.0	100.0

**Table 3:** Surface subsidence during excavation of each part of Section 2.

Distance from midline	Middle hole excavation settlement	Excavation settlement of left and right side holes	Total settlement	Middle cave settlement/total settlement	Side tunnel settlement/total settlement
−25.7	0	−0.58	−0.58	0.0	100.0
−20.7	−0.55	−1.83	−2.38	23.1	76.9
−15.7	−1.69	−2.98	−4.67	36.2	63.8
−10.7	−2.77	−6.52	−9.29	29.8	70.2
−5.7	−6.34	−10.09	−16.43	38.6	61.4
−3.7	−9.12	−11.55	−20.67	44.1	55.9
−1.7	−11.66	−11.12	−22.78	51.2	48.8
1.7	−12.39	−11.46	−23.85	51.9	48.1
3.7	−9.91	−11.87	−21.78	45.5	54.5
5.7	−6.78	−10.64	−17.42	38.9	61.1
10.7	−3.32	−7.18	−10.5	31.6	68.4
15.7	−1.54	−3.51	−5.05	30.5	69.5
20.7	−0.73	−1.99	−2.72	26.8	73.2
25.7	0	−0.63	−0.63	0	100.0

**Table 4:** Surface settlement of each part of Section 3 during excavation.

Distance from midline	Middle hole excavation settlement	Excavation settlement of left and right side holes	Total settlement	Middle cave settlement/total settlement	Side tunnel settlement/total settlement
−25.8	0	−0.44	−0.44	0.0	100.0
−20.8	−0.34	−1.27	−1.61	21.1	78.9
−15.8	−1.27	−2.61	−3.88	32.7	67.3
−10.8	−2.16	−5.32	−7.48	28.9	71.1
−5.8	−4.46	−8.56	−13.02	34.3	65.7
−3.8	−7.53	−10.82	−18.35	41.0	59.0
−1.8	−9.88	−10.58	−20.46	48.3	51.7
1.8	−10.35	−10.26	−20.61	50.2	49.8
3.8	−9.26	−11.01	−20.27	45.7	54.3
5.8	−5.12	−10.64	−15.76	32.5	67.5
10.8	−2.09	−6.02	−8.11	25.8	74.2
15.8	−0.94	−3.11	−4.05	23.2	76.8
20.8	−0.21	−1.49	−1.7	12.4	87.6
25.8	0	−0.57	−0.57	0	100.0

**Table 5:** Accuracy and mean square error of each model.

Network type	The maximum accumulated surface subsidence		The location of the cumulative maximum surface subsidence	
	Average relative error (%)	RMS error	Average relative error (%)	RMS error
BP neural network	3.27	3.87	7.96	21.06
GRNN generalized neural network	4.28	10.00	14.38	24.16
RBF neural network	3.27	10.00	17.38	22.70
Elman neural network	7.85	5.29	11.60	18.71

## Data Availability

The experimental data used to support the findings of this study are available from the corresponding author upon request.
